# Within-Match Performance Fluctuations: Assessment and Observed vs. Expected Extent in Table Tennis

**DOI:** 10.5114/jhk/184275

**Published:** 2024-05-17

**Authors:** Ruizhi Liu, Martin Lames

**Affiliations:** 1 China Table Tennis College, Shanghai University of Sport, Shanghai, China.; 2 School of Medicine and Health, Technical University of Munich, Munich, Germany.

**Keywords:** dynamic analysis, mathematical simulation, double moving average, kurtosis

## Abstract

This study aimed to assess within-match performance fluctuations in table tennis by utilising a dynamic performance indicator, a tailored version of a double moving average. This performance indicator applied to the sequence of wins and losses per rally, modelled a player’s momentary point-winning probability or playing strength. Binomial distribution and Monte Carlo simulations were employed to obtain the expected distributions of double moving averages and their kurtosis. A total of two hundred and eleven single matches from the 2020 Tokyo Olympic Games were examined to characterise the extent of empirical fluctuations and to test for deviations from the expected fluctuations. Results showed that there were large within-match fluctuations (average IQR per match = 0.27). In addition, only one out of the two hundred and eleven matches exhibited a significant deviation from the stochastically expected double moving average distribution. This deviation was observed in the kurtosis of sixteen matches (7.6%). These findings underline the importance of considering within-match dynamic changes when conducting theoretical or practical performance analyses. This consideration should also extend to other performance indicators and various sports games.

## Introduction

A common concept of game sports (invasion games, net games, and striking and fielding games (Read and Edwards, 1992)) is the notion of dynamic interaction processes with emergent behaviour. The constitutive property of this sports group is that performance is realised in permanent confrontation between the two opponents (singles, doubles or teams), each pursuing opposite goals. In net games like table tennis, a rally consists of a series of alternating strokes, where each stroke may be perceived as an “answer” to the former one. Players try to achieve their goals in each stroke: hitting a winning shot or forcing the opponent to make an error (Fuchs et al., 2018).

If one accepts the notion of game sport as dynamical interaction systems (Lames, 2023), there are two significant consequences: first, in game sports, performance should always be evaluated relative to the opponent’s performance, and thus, it cannot be interpreted as a direct indication of the performance level of an athlete. This distinction is worthy of attention when using game analysis as a source for controlling the training process. Second, the dynamic nature of the interaction process gives rise to criticism of the common practice of using static, summative performance indicators to characterise game performances.

Performance indicators describe various aspects of the game (Hughes and Bartlett, 2002), typically expressed as a single figure representing a specific performance aspect's extent, frequency or relative frequency (Sampaio and Leite, 2013). Thus, these conventional performance indicators provide a static, summative description of game behaviour, contrasting the dynamic nature of game sports introduced above.

Performance analysis in table tennis tends to focus on practical aspects such as technical and tactical elements (Fuchs et al., 2018; Malagoli Lanzoni et al., 2014), footwork (Li, 2022), along with fitness and energy expenditure (Zagatto et al., 2018) in the approach of notational analysis. These methods frequently employ one or more static performance indicators to assess players’ specific skills or abilities. Strictly speaking, a static performance indicator like the point winning rate fails to represent the momentary winning rate during the match, for example, when different sets present distinct winning probabilities (WP). O'Donoghue (2009) claimed that performance indicators giving valid and reliable descriptions of performance must encompass within-match dynamics, as a highly fluctuating performance indicator cannot offer a reliable estimation of the underlying performance aspect.

From a practical standpoint, the International Table Tennis Federation (ITTF) continuously revises the rules and regulations of the game to adapt to market-oriented operations. Examples of these changes include increasing the diameter of the ball, shortening the game to an 11-point system per set, altering the materials of the glue and ball, relaxing time limits for bench coaching, and implementing special tournament formats (T2 Diamond, Mixed Team World Cup), etc. Numerous practitioners have acknowledged that these series of reforms have had a considerable impact on players' technical and tactical play. Bin Chen, the personal coach of the latest Grand Slam table tennis player, Ding Ning, commented that these changes significantly intensified matches, creating more fluctuation in on-court situations. In response, he proposed countermeasures, emphasising the cultivation and improvement of players' overall stability as a manifestation of their excellent abilities (Chen, 2007). Xu Yinsheng, the Honorary President of the ITTF, highlighted in a recent interview that upset cases occur frequently in modern table tennis. He underlined the need for not only players, but also researchers to broaden their ideas and innovate their work (Huang, 2023).

Surprisingly, there are limited studies on fluctuations in table tennis within the available literature. Ren et al. (2013) explored variations of players’ psychological states during the match using a cubic spline interpolation. Liu et al. (2017) introduced a fluctuation analysis model based on an improved double moving average (DMA) method. They carefully demonstrated the applicability of this method to table tennis and further enhanced it by employing first a backward moving average and then a forward one, optimally synchronising the match with the DMA representing momentary strength.

The initial introduction of the double moving average (DMA) in sports analysis was in handball (Lames, 2006) and has since been successfully applied in the research of volleyball (Ma, 2019). These studies have proved that the DMA method is a measurable tool to assess within-match fluctuations. It is straightforward to calculate and effectively provides a visual representation of real-time changes in the playing strength of both sides. This intuitive presentation enables coaches and players to quickly observe the ups and downs of performance during the game and make timely adjustments to tactics and strategies. Scientific researchers and practitioners can swiftly identify the distribution characteristics of players' strengths in advantageous, stalemate and disadvantageous periods in a specific game. Through long-term or multi-game observation, they can also discern patterns or essential features of players’ performance variations. This information could serve as a guide for further in-depth analyses (Liu et al., 2017).

In contrast, previous studies have been primarily descriptive in nature. A critical area of improvement involves stochastic modelling of the expected distribution of DMAs within a match and comparing it with empirical distributions. This comparison may help answer questions about the nature of within-match fluctuations: are they purely random, or do specific determinants influence these deviations? Besides this question, which is more of a theoretical interest, understanding the expected and actual magnitude of within-match fluctuations holds practical significance for game analysis as it provides a framework for reconstructing and interpreting performances. Practical match analysis aiming at generating hints for training by identifying strengths and weaknesses in a match (Lames, 2023) has to be conducted differently when there are sizeable within-match performance fluctuations or when constant behaviour may be assumed. Fluctuations add a new dimension to the interpretations of match behaviour.

Therefore, the aims of our study were threefold: (1) a representative sample of top-level table tennis matches was analysed using DMAs to provide descriptive statistics on DMAs in actual top-level men’s and women’s table tennis; (2) the expected distribution of DMAs was modelled using known stochastic properties of table tennis matches, e.g., the overall winning probability and the binomial distribution of k successes in a series of n rallies given the overall winning probability. In addition, relevant aspects of the expected shape of the DMA distribution (flatter or steeper distribution, i.e., the kurtosis) were modelled stochastically; (3) based on the first two steps, a comparison between observed and expected DMA distributions was carried out, providing insight into whether fluctuations could be attributed to purely stochastic effects.

## Methodology

### Participants

The sample covered all matches in Men’s and Women’s Singles (best of seven sets) and all single matches in Men's and Women's Teams (best of five sets) from the 2020 Tokyo Olympic Games (n_men_ = 105; n_women_ = 106; one male player DNS). There were 76 male and 83 female players, each from 44 participating countries, included in the study. Their rankings were from 1^st^ to 1543^th^ and 1^st^ to 1110^th^, respectively, according to the Men’s and Women’s singles ranking list as of 20^th^ of July, 2021, issued by the ITTF.

### Data and Observer Agreement

Nationalities of all participants and accurate score data for each match were sourced from the results book of the 2020 Tokyo Olympic Games available on the online Olympic World Library. The nationality information provided in the results book was cross-verified against the official data from the ITTF website, and no discrepancies were found. The inter-rater reliability test was conducted with regard to the results per rally using the Kappa statistic on a randomly selected five percent of the matches (11 matches, 6 men’s and 5 women’s, 1146 rallies) from the sample. All scores in these selected matches were observed and recorded by a German B-licenced table tennis coach working at a Bundesliga club. The consistency was rated as “very good” (Altman, 1990) with the coefficient of 0.998.

Based on the 11-point system, the scores were converted into 1 (score) or 0 (loss) point by point from the winner’s perspective. Due to the principle of the DMA method and the binomial distribution characteristic of its calculation indicators (1 or 0), the output data from the winning side were symmetrical with those of the losing side at the 0.5 level. Therefore, all data in this study was calculated from the winner's point view.

### Independent Variables

#### Match Categories

Matches were categorised into three groups, i.e., matches between high-ranked, low-ranked, and mixed, to check for the impact of the match performance level and homogeneity on within-match fluctuations. The world ranking of 35^th^ was set as the division point since this would minimise the difference in the number of matches in each group with respect to gender, in order to minimise statistical errors arising from sample sizes.

#### Improved Competition Performance

The results of CP for both the winner and the loser were symmetrically distributed above and below 11, with a minimum of 0 (indicating all sets lost with 0–11) and a maximum of 22 (representing all sets won by 11–0). However, the traditional CP formula (Zhang et al., 2013) assigns equal weight to sets ending with scores like 11–9, 12–10, 13–11, …, although dominance is decreasing in this sequence. Thus, an improved formula was developed to address this drawback:


$$CP=22-\sqrt{\sum_{i=1}^{n}\frac{{\left(SCP_{i}-11\right)}^{2}}{n}}\;\left(1\right)$$

$$\left\{\begin{array}{c} 11+{\text{Setpoints}}_{loser,i},\;\left({\text{Setpoints}}_{winner,i}=11\right)\\ 11\pm \frac{1.25}{{\text{Setpoints}}_{winner,i}-11},\;\left({\text{Setpoints}}_{winner,i}>11\right) \end{array}\right.\left(2\right)$$

Note: CP represents improved competition performance, n is the number of sets in the match, i represents the i^th^ set in the match, and SCPi represents the improved competition performance for the i^th^ set.

### Assessment of Within-Match Fluctuations

#### Rationale of DMA

**Figure 1:**
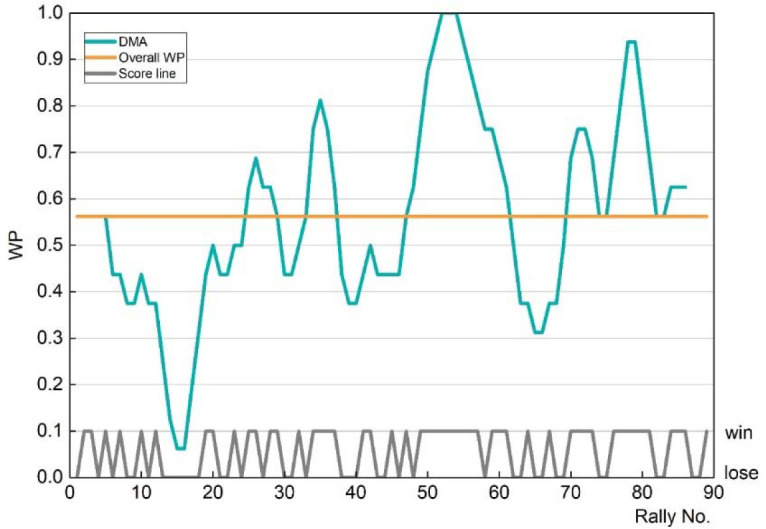
Quantification of within-match performance in table tennis by overall winning probability, results per rally and the DMA.

Modelling within-match fluctuations requires a time-sensitive indicator that represents a compromise. On the one hand, it should not be overly dependent on very concise performance fluctuations, e.g., the outcomes of two consecutive rallies in table tennis. On the other hand, the indicator should be sensitive enough to capture performance developments occurring during a reasonably short period, e.g., 5 to 10 rallies. A DMA can be seen as a dynamic representation of within-match performance in sports. As depicted in Figure 1, it takes an intermediate position between the highly variable outcomes seen in individual rallies, as is common in table tennis, and the completely static overall winning probability.

The moving average represents a straightforward smoothing method that calculates the sequential average of a specific number of terms from a time series, thereby reflecting a smoothed general trend. This technique helps filter out short-term and random influences. In the literature, we find the simple moving average of a particular length n (formula 3), weighted moving averages and the double moving average (Hua, 1995).


$$M_{i}=\frac{y_{i}+y_{i-1}+\cdots +y_{i-N+1}}{N}\text{ (3)}$$

A double moving average (formula 4) takes a time series of simple moving averages as its foundation for calculating a secondary (double) moving average. This approach enhances the level of smoothing while preserving the benefits of a simple moving average, i.e., it relies on a relatively small number of data points, allowing it to capture local developments effectively (Hua, 1995).


$$DMA=M_{i}^{\left(2\right)}=\frac{M_{i}+M_{i-1}+\cdots M_{i-N+1}}{N}\;\left(4\right)$$

### Observed DMA Distribution

Its application in table tennis has been proven in detail by Liu et al. (2017) and listed as a method of performance analysis in table tennis in the paper of Fuchs et al. (2018). In this study, the authors applied this improved DMA (Formula 3 & 5). That is, each rally was assigned a value of 1 for a win and 0 for a loss. The moving window was set to four rallies as this is the optimal choice between stability and flexibility (Liu et al., 2017). The first moving average (MA) was calculated in the forward direction (formula 3), while the second one (DMA) was computed as a backward moving average of four sequential MAs (formula 5), thus optimally synchronising the match with the DMA representing momentary strength. A line chart depicting the DMA across the rallies of a match illustrates the ups and downs of performance during the match (Figure 1, line “DMA”).


$$DMA_{i}=\frac{M_{i}+M_{i+1}+M_{i+2}+M_{i+3}}{4}\text{ (5)}$$

### Expected DMA Distribution

The algorithm for calculating a DMA leads to a limited set of 17 discrete values, i.e., 0, 0.0625, 0.125, 0.1825, …, 0.9375, 1. These are the only discrete values obtained for a DMA distribution using the abovementioned procedure. Moreover, an alternative description for a DMA is taking it as a weighted sum of the result variable (x = 0 or 1) of seven consecutive rallies:


$$DMA_{i}=x_{i-3}+2x_{i-2}+3x_{i-1}+4x_{i}+3x_{i+1}+2x_{i+2}+x_{i+3}\;\left(6\right)$$

**Figure 2:**
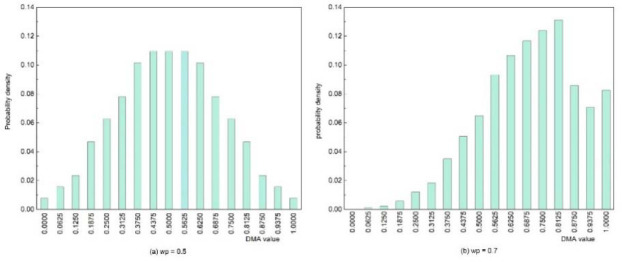
Expected densities of DMA distributions for winning probability of 0.5 (a) and 0.7 (b).

The expected DMA distribution can be derived through the following five steps:

1. Enumerate all possible sequences of wins and losses in a sequence of seven rallies, resulting in 128 possible sequences (since there are two outcomes per rally).
2. Utilise the binomial distribution to determine the probability of achieving 0,1,2...,7 wins in seven rallies, given the overall point-winning probability. Assign these probabilities to each of 128 possible sequences.
3. Calculate the DMA for each sequence using formula 5.
4. For each of the 17 possible values of the DMAs, compute the sum of the probabilities obtained in step 2.
5. Plot the resulting distribution of the DMAs given a certain point-winning probability, as a column diagram (Figure 2).

### Expected Kurtosis of DMA Distributions

To assess deviations in the empirically observed DMA distributions within a match, a second method employs the concept of kurtosis. The kurtosis is a statistical measure that describes the sharpness of a distribution’s peak in relation to a normal distribution. Its advantage lies in not relying on assumptions on the distribution, such as assuming a binomial distribution for the number of wins in a sequence of seven rallies. From the perspective of within-match fluctuations, the kurtosis of a DMA distribution expresses its steepness or flatness (“peak”). Specifically, a distribution steeper than expected suggests that the behaviour is more stable than expected, while a flatter distribution signifies larger within-match fluctuations. Thus, by comparing the kurtosis of the empirical and expected DMA distribution, one can test for relevant deviations without relying on the assumption of a particular distribution, in a non-parametric way.

In order to test whether an empirical kurtosis of DMAs in a specific match falls within or outside the confidence limits of the expected kurtosis, the distribution of the expected kurtosis is simulated. Statistical testing is founded on a Monte Carlo simulation of the expected distribution of the DMA kurtosis for a specific match. The procedure for one match comprises six steps:

1. Using a random number generator, a sequence of 1 million 0s and 1s is created based on the given overall point-winning probability of this match.
2. The DMA is then calculated for this sequence of 0s and 1s.
3. Given the number of rallies “n” in this match, the DMA-time series is segmented into pieces named “Monte Carlo matches”, each with a length of “n” (typical number of rallies: 100; typical number of Monte Carlo matches: 10,000).
4. For each of these Monte Carlo matches, the kurtosis of the DMAs is calculated.
5. The mean and standard deviation of this kurtosis distribution are calculated.
6. A two-sided z-test assuming a normal distribution of approximately 10,000 simulated kurtosis values is run, testing whether the empirical kurtosis of the match under scrutiny falls within the 95% confidence interval of the Monte Carlo simulated kurtosis distribution.

This six-step procedure yields a statistical result that indicates whether the empirical kurtosis of the DMAs in a match significantly deviates from the mean kurtosis one would expect for the DMAs obtained for the overall point-winning probability of the match. In other words, it reveals whether within-match fluctuations significantly exceed or remain below their expected values.

**Table 1:** Match categories with frequencies of matches.

Group	Male		Female		Total	
	Count	%	Count	%	Count	%
<35 vs. <35	43	40.95	23	21.70	66	31.28
<35 vs. >35	34	32.38	45	42.45	79	37.44
>35 vs. >35	28	26.67	38	35.85	66	31.28
Total	105	100.00	106	100.00	211	100.00

Note: <35 and >35 represent the world ranking in or outside the top 35^th^ places, respectively.

### Statistical Data Analysis

The descriptive statistics of table tennis singles matches at the 2020 Tokyo Olympic Games were calculated from the perspective of the winners. This included the calculation of mean, standard deviation, maximum, minimum, and median values for DMA, WP, and CP for both male and female players. The Mann-Whitney U-test (*p* < 0.05) was used to test the gender-related differences of the above indicators and the differences in kurtosis, CP, WP, as well as fluctuations between each pair of ranking groups (*p* < 0.05 with Bonferroni correction). The Kruskal-Wallis H-test (*p* < 0.05) was used to test the difference between the three ranking groups. The Kolmogorov-Smirnov (K-S) test was used to compare the observed and expected DMA distributions. Significant deviations of the observed kurtosis from the expected ones were tested by a z-test employing the mean and the standard deviation of the expected distribution.

## Results

### Descriptive Statistics on Observed Values

Table 2 presents the descriptive data for table tennis singles matches at the Tokyo Olympic Games. It includes the number of players, mean, standard deviation, maximum, minimum, and median values of WP and CP for both male and female players. Additionally, Z and *p* values are presented indicating gender differences. As can be seen from the table, the mean values for male players were significantly lower than those for female players on DMA, WP, and CP. The kurtosis of male players was also lower than of female players, but this difference was not statistically significant. The Kolmogorov-Smirnov test revealed that the values of DMA were not normally distributed (n = 211, D = 0.075, *p* = 0.006).

**Table 2:** Descriptive statistics for the mean of the DMA, WP, CP and kurtosis of the DMA and U-Test results for mean differences between male and female players.

				N		Mean		Std		Max	Min	Median	Z	*p*
**DMA**	Male		105		0.563		0.069		0.748		0.358	0.557	−3.313	0.001
	Female		106		0.595		0.069		0.807		0.471	0.581		
**WP**	Male		105		0.577		0.061		0.772		0.474	0.564	−2.872	0.004
	Female		106		0.601		0.066		0.805		0.486	0.588		
**CP**	Male		105		13.119		2.116		18.464		8.760	13.014	−2.032	0.042
	Female		106		13.747		2.190		19.292		9.442	13.500		
**Kurtosis**	Male		105		−0.429		0.457		1.187		−1.339	−0.446	−1.470	0.141
	Female		106		−0.494		0.497		1.141		−1.307	−0.575		

The H-tests indicated that there was no significant difference in the following indices among the three different performance categories (<35 vs. <35: n = 66; <35 vs. >35: n = 79; >35 vs. >35: n = 66): mean of DMA (H = 0.512, *p* = 0.774), WP (H = 0.489, *p* = 0.783), CP (H = 0.236, *p* = 0.889), and kurtosis (H = 0.063, *p* = 0.969).

Rank correlation analyses revealed that the mean of DMA had very positive correlations with WP (*ρ* = 0.945), CP (*ρ* = 0.902), and score difference (*ρ* = 0.893) at the 0.01 level. However, there was no significant correlation with ranking difference (*ρ* = 0.125).

Within matches, the DMA values of winners touched the upper limit of 1 in 109 of the 211 matches investigated (51.67%), while the lower limit of 0 was met by winners in 31 matches (14.69%). The average standard deviation of DMA per match was 0.194, and an average IQR of 0.270 was found. These descriptive statistics for DMA within matches can be interpreted as an expression of high within-match fluctuation.

### Observed and Expected DMAs

**Figure 3:**
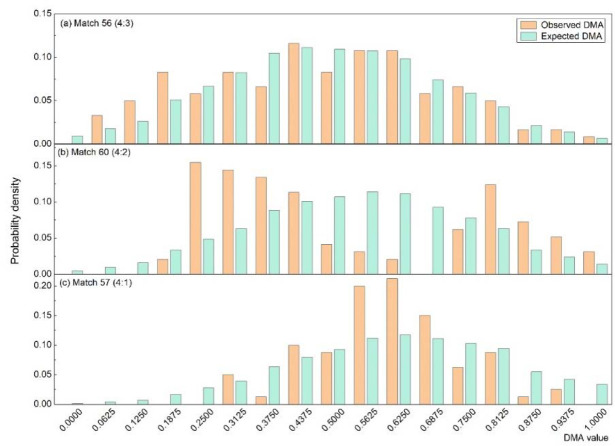
Comparison of the observed and expected DMA distributions for three matches.

Figure 3 displays three diagrams containing examples of empirical and expected distributions of DMA in three Men's Singles Matches. These examples were chosen to illustrate matches with: a) a DMA distribution similar to the expected one; b) a DMA distribution with higher-than-expected fluctuation (i.e., more phases with less and more success than expected); c) a “steeper” distribution indicating a more stable DMA than expected. The K-S tests resulted in a) d = 0.062, b) d= 0.204, and c) d = 0.141, respectively.

A significant difference in the distance (d value) of K-S tests per match between the cumulative observed and the cumulative expected distribution was found in only one male match.

### Observed and Expected DMA Kurtosis

The U-test revealed no significant difference between observed and expected kurtosis for the sample (Z = −0.036, *p* = 0.972). The z-tests were further conducted match by match. There were sixteen (7.58%) matches that exhibited a significant deviation of the observed kurtosis from the expected one. The proportions for male (7 matches; 6.67%) and female (9 matches; 8.49%) players were similar.

## Discussion

In this study, we introduced a method for assessing the within-match fluctuations in table tennis matches using the DMA. The DMA represents the momentary performance of the winner (1−DMA: the value for the loser) in a match, representing a compromise between a static variable (in this case, the point-winning probability of the winner) and the rally-by-rally success-failure sequence. This approach offers some conceptual advantages, as illustrated in Figure 1.

The within-match fluctuations of performance were characterised using three different approaches. First, descriptive statistics of DMAs for each match were analysed. We found that in 51.6% of all matches, the DMA reached the value of 1, signifying a streak of 7 consecutive wins, while in 14.6% of all matches, the DMA of the winner touched the lower limit of 0, meaning that winners experienced at least one streak of 7 lost rallies. Beyond these extreme events, the volatility of the DMA was further assessed by examining average dispersion statistics across 211 matches. The average standard deviation of the DMA was 0.193, which resulted in a coefficient of variation of 33.41%. The average interquartile range was determined to be 0.270. This means that typically, 50% of all rallies in a match exhibited a range of more than 0.587 in winning probability within the course of the match. These descriptive findings strongly suggest that momentary winning probability and playing strength display significant volatility during a match, indicating high levels of within-match fluctuation in performance.

It is evident from Table 2 that female winners, on average, outperformed male winners in the DMA, WP and CP. We could deduce that this resulted from the higher performance fluctuations in male players during the game, in other words, female players’ performance tended to be steadier. The following studies could well serve as an empirical basis for this inference.

Tamaki et al. (2017) found that male players exhibited higher scoring as well as losing rates than female players in each of the eight shot categories across seventy Olympic matches. Out of these, five and six comparison groups respectively, showed statistical significance. The effectiveness, representing the scoring or losing tendency at the i-th shot, was significantly different in four shot categories, with men being lower than women in three of them. Additionally, Zeng (2023) also observed that female players had higher hitting efficiency than male players through video kinematic analysis.

Pradas de la Fuente et al. (2023) asserted that male and female players exhibited different game dynamics following a detailed stroke-by-stroke analysis of twenty-four high-level matches focusing on technical-tactical actions. One of the manifestations of this was that more winning actions were performed in the men’s than in the women’s match. In spite of that, men also performed more losing technical actions, particularly when applying the forehand and backhand flips, which were identified as the predominant losing techniques.

These findings further confirm that the DMA yields results similar to the most commonly used performance indicators for match dominance, such as WP and CP. Ranking difference, an indicator that reflects the disparity in strength between two players to some extent, was also expected to lead to significant findings. However, it showed no significant correlation with the DMA (n = 211, *p* = 0.070). We can speculate that this lack of the relationship may be influenced by the ranking system, a topic which we discuss in the following paragraph, along with the Olympic qualification system.

The second method for characterising within-match fluctuations involved comparing the observed distributions of the DMA with the expected ones. The expected distribution was only dependent on the overall winning probability of a particular match, assuming a binomial distribution for the number of wins in a sequence of seven rallies (Figure 2). The K-S tests revealed significant differences in only 0.47% of the matches. This suggests that the substantial DMA fluctuations identified in its descriptive statistics can be attributed to stochastically expected fluctuations to a large extent.

The third approach to characterise within-match fluctuations relies on the kurtosis of the DMA distributions. High kurtosis corresponds to a “steep” distribution tightly centred around its mean. In a table tennis match with high kurtosis in its DMA distribution, there are many phases with winning probabilities similar to the average, indicating low within-match fluctuations. Conversely, low kurtosis results in a “flatter” or bi- or multi-modal distribution, signifying a high rate of within-match fluctuations. The results showed that only 7.6% of the matches exhibited empirical kurtosis values outside the 95% confidence interval of the expected kurtosis. It reveals that the within-match fluctuations, as characterised by kurtosis, were mostly in line with what was stochastically expected.

In contrast to the present research on within match fluctuations, the study conducted by Hughes et al. (2015) may be perceived as mere processing of scorelines, which exhibited a relatively simple format for presenting results. Although it is capable of illustrating the high and low performing phases in a match, it lacks clear assessment criteria.

While the research approach of Ren et al. (2013) differs from the approach in this work, the underlying principles are comparable. Their comparative analysis of inhomogeneous players and individual comparative analyses of different phases of the game revealed significant individual differences in the performance of top-level table tennis players. They observed players experiencing collapses in major tournaments, particularly against foreign top players and at different match phases. Their discoveries confirm the conclusions of this study from a psychological perspective.

The study conducted by Russomanno et al. (2021) in handball is most similar to the current one. Russomanno et al. (2021) classified the momentary strength (DMA) of all twenty-four teams into ten bins and compared the distribution in each bin. Significant differences were found in eight bins, with all twenty-four teams hitting the lowest bin and eighteen rising to the highest, irrespective of their final position.

In classical table tennis analytical theories, only within rally fluctuations were of interest, as seen in the evolution of Three-Phase Evaluation Theory. This method is credited with playing an important role in supporting the Chinese Table Tennis Team's repeated championships over the past three decades, which remains in use today (Xu, 2018). The intrinsic nature of this theory is a mapping of within-rally fluctuation. In practice, scholars have made diverse improvements to it, such as the four-phase method, the two-round- five-phase method, the dynamic three-phase method and various modified multi-phase methods. These enhancements aim to produce the results more “realistic”, “precise”, “individual” or “sensible to actual circumstance” (Zhang et al., 2018). The emergence of these methodologies not only supplements traditional static indexes in dynamic performance response, but also indirectly reflects the ever-changing nature of players' performance, which makes the researchers keep seeking for the optimal solution.

The findings of the aforementioned researchers align perfectly with the results of this study, reinforcing the notion that a player's performance can exhibit significant variability between and within matches and rallies. Whether experiencing strong or weak phases during a match, players should always maintain self-belief and striving for optimal success. Coaches should give full consideration to the personalised and spatial features unique to different genders and individuals when formulating training programs and tactical strategies, and carry out targeted enhancement training for specific items. Researchers can contribute by: (1) using this method as a quick approach to assess players' momentary strength, providing real-time feedback when necessary; (2) consulting the method proposed by Liu et al. (2017) to categorise advantageous, stalemate, and disadvantageous periods, conducting in-depth analysis, including, but not limited to technical and tactical assessments; (3) exploring other research emphases or performance indicators for within match dynamics on one player or a sample of players, including the mentioned three periods.

## Limitations

The first limitation of the study considers the sample of table tennis matches employed. At the Tokyo Olympic Games, there were 1543 positions within the ranking span among all players. This is a result of the unique qualification system, which is quite unusual compared to other tournaments. The ranking positions were determined based on points accrued from a player's best eight ITTF events over the preceding twelve months, by the ranking rules at that time. The more ITTF tournaments or platinum series tournaments a player participated in (which offer the highest bonus points), the more likely he or she was to accumulate higher points. This potentially produces some overlap or interference between different performance level categories. Nonetheless, it is emphasised once more that the rankings may not precisely reflect players’ true strength, and real-time performance and match-to- match fluctuations are some of the most objective indicators of players’ performance.

Another potential concern relates to the stochastic assumptions used when modelling the expected distribution of the DMA. The fundamental probability of winning a specific number of rallies in a series is modelled under the assumption of independent trials that follow a binomial distribution. Notably, the first assumption of independent trials does not consider any sort of “streakiness”, i.e., that outcomes of former rallies may elicit a tendency toward similar outcomes in future ones. If such streakiness had a strong impact, one might expect that a series of rally outcomes with positively auto-correlated results would exhibit lower variability than independent outcomes. Yet, it is crucial to note that this particular observation is not supported by empirical results. Moreover, when employing a non-parametric method involving kurtosis, the outcomes remained comparable. These findings suggest that the assumptions used in the modelling appear to be adequately justified.

A final point of contention pertains to the method used to assess the agreement between observed and expected DMAs and kurtosis distribution. It is important to emphasise that, in the instances of significant rejections, the empirical findings do not conform to the tested distributions. Nevertheless, it is worth noting that in the vast majority of cases, it has been demonstrated that there is no significant deviation from the expected distributions. This can be considered the statistical evidence required to substantiate the conclusions drawn in the present study.

Another intriguing question arises: can fluctuations be observed for performance indicators beyond playing strength? A supplementary investigation was conducted to explore this. One of the most widely recognised performance indicators in table tennis is technique effectiveness (TE,(Zhang et al., 2013). TE combines the scoring rate with the usage rate to assess the technical and tactical strength of players in each phase (TE_1,3_, TE_2,4_, TE_>4_) or throughout the entire match (TE overall). This dynamic performance indicator was subjected to analysis by evaluating traditional TE_1,3_ and TE overall values with a moving window of 20 rallies in an illustrative match (2018 Swedish Open: ITO Mima vs. LIU Shiwen; 4–3 (12:10,7:11,7:11,5:11,13:11,11:4,11:8)).

**Figure 4:**
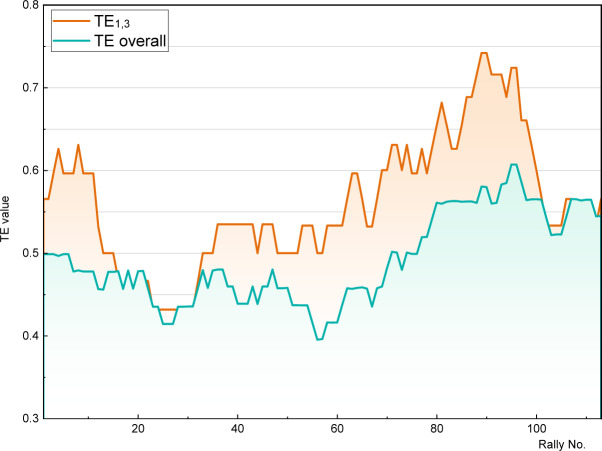
Within-match dynamics of traditional performance indicators TE_1,3_ and TE overall.

Figure 4 shows the course of TE_1,3_ and TE overall for the match. It can easily be perceived that these traditional performance indicators also exhibit significant fluctuations within the match. Furthermore, it is clear that the statistical norms typically associated with specific levels of static performance indicators apply only to certain phases of the match. Taking dynamics into account also means that a single label may not accurately reflect a player’s “true” performance throughout the match.

## Conclusions

This study demonstrates that the momentary playing strength in table tennis shows large within-match fluctuations, primarily of a random nature. From the perspective of theoretical performance analysis, this confirms the general notion of game sports as dynamic interaction processes with emergent behaviour. Performance analysts are highly recommended to take within-match fluctuations into account when conducting comprehensive assessments of the game. From a practical perspective, this may be seen as a piece of advice for players and coaches that players' performance is characterised by high levels of randomness and fluctuations. Therefore, coaches and players should maintain a state of alertness and proactivity to adapt effectively to these dynamic variations.

There is every reason to believe that these findings hold for other performance indicators and other game sports as well. It underlines the importance of exploring within-match fluctuations of performance indicators, with the proposed DMA method as a valuable tool. This practice should be incorporated as a regular component of theoretical and practical performance analysis.
